# Impact of High-Flow Nasal Cannula on Arterial Blood Gas Parameters in the Emergency Department

**DOI:** 10.7759/cureus.10516

**Published:** 2020-09-17

**Authors:** Emre Şancı, Feride Ercan Coşkun, Basak Bayram

**Affiliations:** 1 Emergency Medicine, Kocaeli Derince Training and Research Hospital, Kocaeli, TUR; 2 Emergency Department, İzmir Çiğli Training and Research Hospital, Izmir, TUR; 3 Emergency Department, Dokuz Eylul University, Izmir, TUR

**Keywords:** high flow nasal cannula, emergency department, hypercapnia, arterial blood gas

## Abstract

Background: High-flow nasal cannula (HFNC) oxygen is becoming an integral part of respiratory failure management. Effects of HFNC on arterial blood gas (ABG) parameters especially partial carbon dioxide (PaCO2) require further investigation to provide insight into the efficacy and safety of the treatment.

Methods: Acute respiratory failure patients with sequential ABG parameters before and after initiating HFNC between June 2015 and June 2017 were analyzed in a tertiary academic center. Patients' baseline characteristics were evaluated and sequential ABG changes were compared and subgrouped as chronic obstructive pulmonary disease (COPD), respiratory acidosis, hypercapnia, and high lactate.

Results: A total of 120 patients were enrolled in the study. There was a significant difference between the mean partial pressure of oxygen in arterial blood (PaO2), lactate, and peripheral oxygen saturation (SpO2) values between sequential ABGs after HFNC (P <0.001). In the COPD group (n=32), there was a significant difference between initial ABG means of PaO2, lactate, and SpO2 values and sequential ABG means (p<0.001). Hypercapnic patients PaCO2 levels were significantly lower after HFNC (p<0.001), while in the COPD group there was no significant change in PaCO2 values (p=0.068).

Conclusions: Treatment with HFNC produced improvement of blood gas parameters in subjects with acute respiratory failure in the emergency department (ED). These results suggest that HFNC can be used in hypercapnic patients as well as hypoxemic patients. Further randomized controlled studies required to establish the impact of HFNC in the ED.

## Introduction

High-flow nasal cannula (HFNC) oxygen has been used as an adjunct treatment of patients with acute respiratory failure in the emergency departments (ED) and intensive care units. HFNC delivers humidified and heated air-oxygen mixture at flow rates up to 60 L/min, through a nasal cannula. Physiological short term effects of HFNC include reducing partial carbon dioxide (PaCO2) as well as improving oxygenation. While supplemental oxygen administration is still considered the first-line treatment of acute respiratory failure (ARF), HFNC provides advantages such as high-flow rates parallel to patients’ inspiratory flow creating positive end-expiratory pressure (PEEP) [1,2], reducing anatomic dead space [3], providing humidified and heated air which improves CO2 excretion [4], sputum clearance, and mucociliary motion [5].

HFNC becoming a ubiquitous treatment and might be used as initial respiratory support instead of conventional oxygen therapy or non-invasive ventilation in certain scenarios such as non-compliance to non-invasive ventilation and inadequate oxygenation via conventional methods. HFNC utilization results in the delivery of higher rates of flow during oxygenation, therefore, naturally occurring mismatch of flow rate difference via supplemental oxygen therapy is reduced. Therefore, HFNC utilization is becoming increasingly common in EDs for ARF patients.

ARF includes not only hypoxic patients but also hypercapnic patients or patients prone to hypercapnia. High rates of oxygen therapy might increase the tendency to hypercapnia and its association with the poor outcome [6]. Baseline hypercapnia can be seen on admission to ED with chronic obstructive pulmonary disease (COPD) exacerbation and chronic hypercapnic COPD patients. Patients admitting to EDs with acute respiratory failure needs conventional oxygen therapy or invasive/non-invasive mechanical ventilation depending on the severity of their condition. As an alternative to conventional treatment for patients who require increased oxygenation, HFNC can be utilized. In this study, we aimed to examine the changes in arterial blood gas (ABG) parameters with HFNC in ED which would provide insight into the safety and efficiency of the treatment.

## Materials and methods

Study design and setting

This study was conducted as a retrospective analytical study in a tertiary academic center with an annual census of 125.000 patients. The Institutional Ethics Committee of Dokuz Eylul University approved the study (3572-GOA). The authors evaluated charts of patients treated with HFNC between June 2015 and June 2017. HFNC was delivered using an Optiflow™ nasal interface connected to AIRVO humidifier (Fisher & Paykel Healthcare, Auckland, New Zealand). In our ED, HFNC treatment protocol dictates that flow is initiated 30lt/min and later titrated as based on sequential ABG results and oxygen saturation and patient clinical status.

Study population

Patients in the ED with ARF who received HFNC between June 2015 and June 2017 were evaluated for the study. ARF for this study is defined as partial pressure of oxygen in arterial blood (PaO2) <60mmHg on room air in patients admitting to ED with dyspnea. Sequential ABGs obtained before and after the onset of HFNC were examined. Exclusion criteria include mechanical ventilation, age less than 18, no initial ABG, lack of documented sequential ABGs, and time interval > 12 hours between sequential ABGs.

Data analysis

Statistical analysis was performed using SPSS version 20 (SPSS, Inc, Chicago, IL) software. R was used for paired dot plots. Categorical variables were presented as numbers and percentages. The appropriateness of the data to the normal distribution was examined by the Kolmogorov-Smirnov test. Continuous variables were reported as the median and interquartile range (IQR) (25th-75th interquartiles). The mean of the normal distribution of numerical values was compared with the paired t-test and the mean of the non-normally distributed numerical values were compared by using the Wilcoxon test. The change in categorical variables in the dependent group was compared using the McNemar test. P values below 0.05 were evaluated as statistically significant results.

## Results

**Figure 1:**
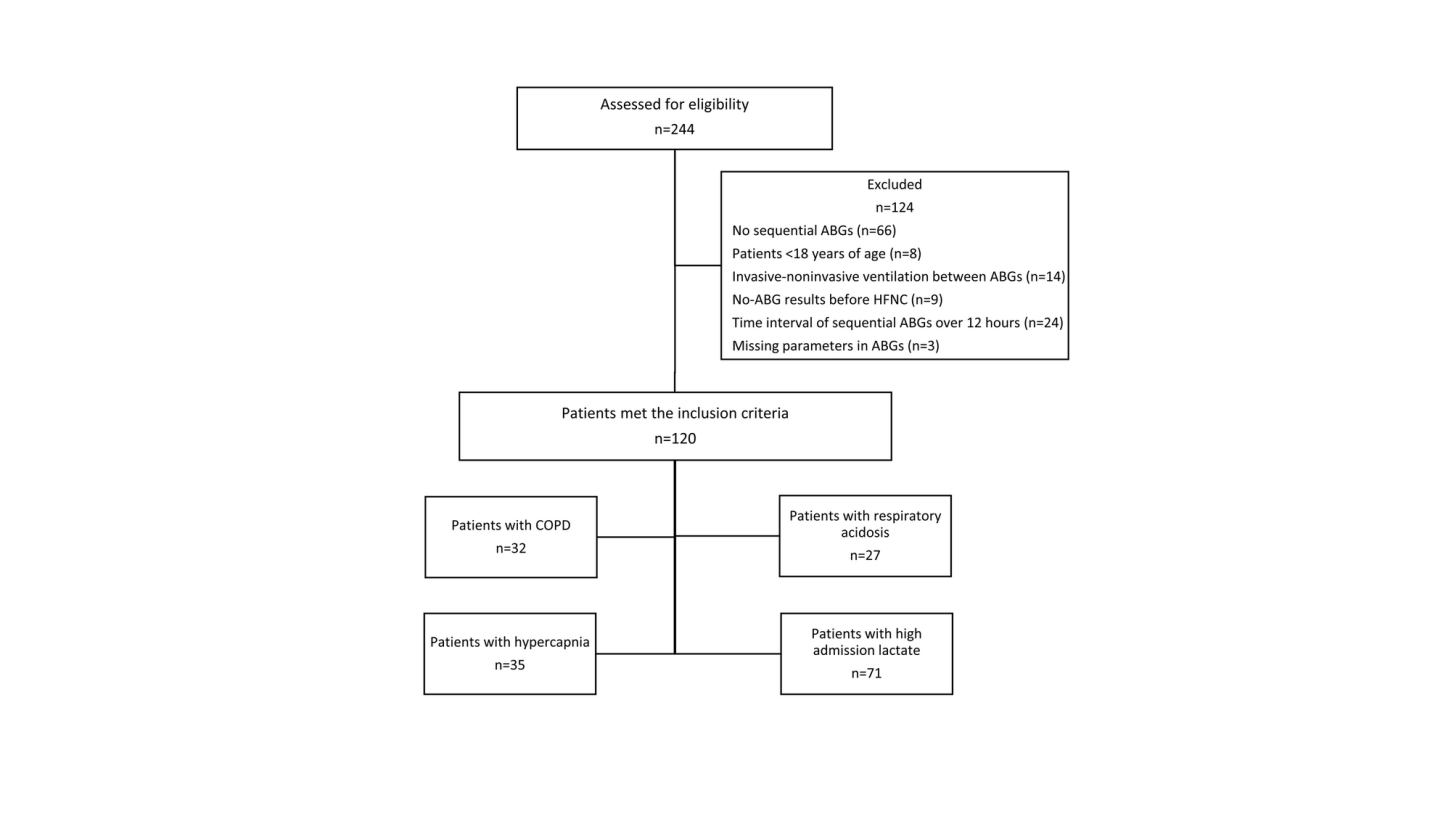
Flow diagram of the study ABG: arterial blood gas; HFNC: high-flow nasal cannula oxygen; COPD: chronic obstructive pulmonary disease.

A total of 244 patients were assessed for eligibility, 120 met the study criteria and were enrolled in the study (Figure 1).

Patient characteristics

Patient characteristics are detailed in Table 1. The median age was 73.5 (IQR: 64.5-82). The most common etiology of using HFNC was pneumonia - 61/120 (50.8%).

**Table 1:** Patient characteristics

Age (median-interquartile range)	73.5 (64.5-82)
Gender	76 (63.3%)
Male	76 (63.3%)
Female	44 (36.7%)
Comorbidities	
Interstitial pulmonary disease	6 (5%)
History of pneumonia	2 (1.7%)
Chronic cardiac failure	29 (24.2%)
Coronary artery disease	3 (2.5%)
Chronic obstructive pulmonary disease	32 (26.7%)
Lung cancer	20 (16.7%)
Ongoing malignancy (Other)	9 (7.5%)
History of pulmonary embolism	7 (5.8%)
Obstructive sleep apnea syndrome	2 (1.7%)
Bronchiectasis	1 (0.8%)
Etiology of respitory failure	
Pneumonia	61 (50.8%)
Congestive heart failure	17 (14.2%)
Cancer	29 (24.2%)
Chronic obstructive pulmonary disease	18 (15%)
Interstitial pulmonary disease	6 (5%)
Pulmonary tromboembolism	7 (5.8%)
Pulmonary vasculitis	1 (0.8%)
ED Outcome	
Admitted to general ward	51 (42.5%)
Admitted to ICU	30 (33.3%)
Discharged	6 (5%)
Discharged against medical advice	6 (5%)
Death	17 (14.2%)

Changes in ABG parameters after HFNC

**Figure 2:**
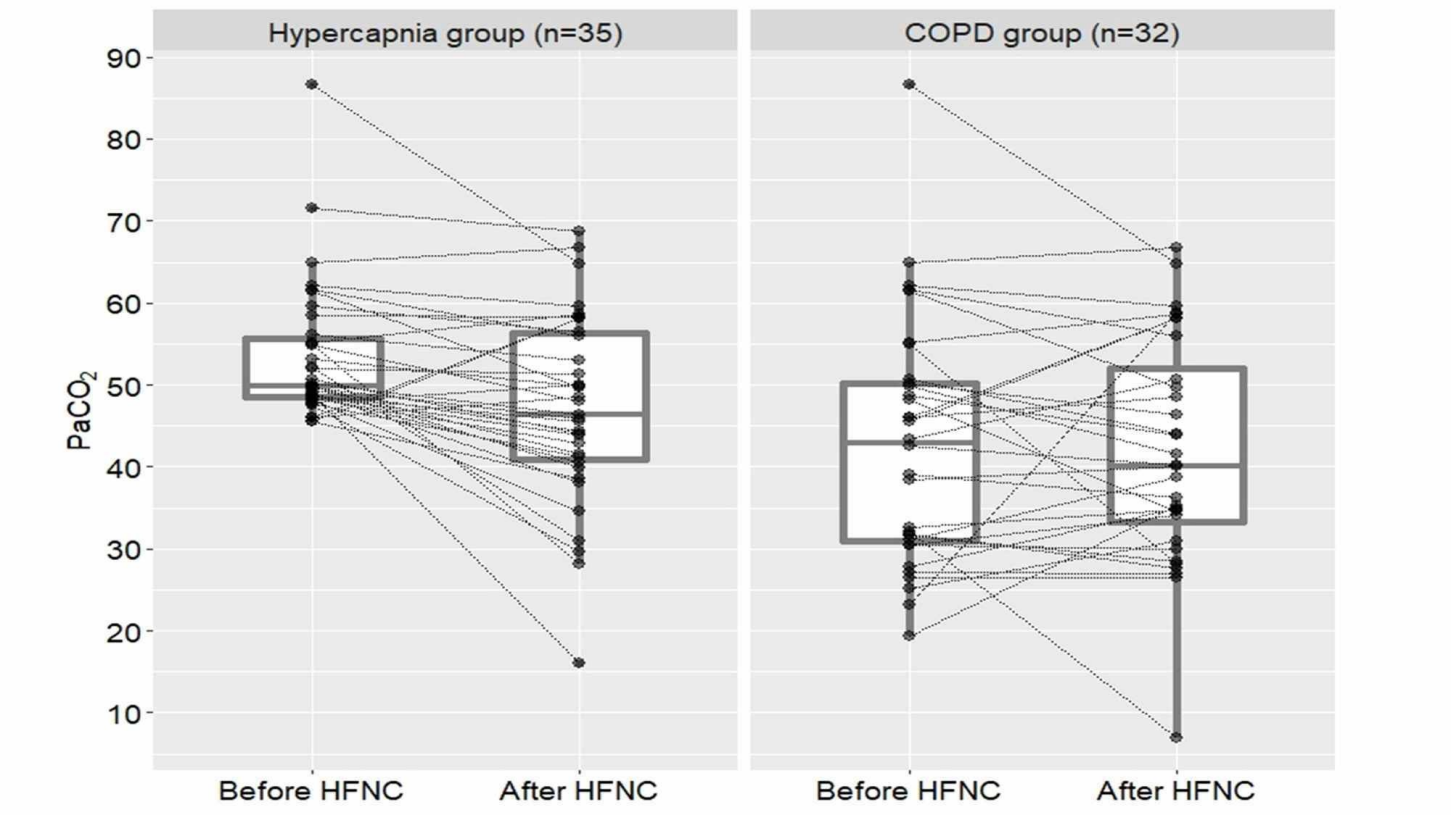
PaCO2 changes of patients hypercapnia and COPD groups in paired dot-plot HFNC: high-flow nasal cannula oxygen; COPD: chronic obstructive pulmonary disease; PaCO2: partial carbon dioxide.

The median time between initial and sequential ABGs was 2.75 hours (IQR: 1.5-6). In all 120 patients, there was a statistically significant difference between the mean PaO2, lactate, and peripheral oxygen saturation (SpO2) values of the initial and sequential ABGs following HFNC utilization (p<0.001). Additionally, HFNC was not found to have an impact on lowering PaCO2 levels (p=0.47) in all patient group. In the COPD group (n=32), there was a significant different between the initial PaO2, lactate, and SpO2 and sequential ABG values (p<0.001) while there was no significant change in PaCO2 (p=0.068). In the hypercapnia group (n=35), PaCO2 levels were significantly lower after implementing HFNC (p<0.001). Patients' ABG parameters are summarized in Table 2. PaCO2 changes in the hypercapnia and COPD groups are displayed in Figure 2.

**Table 2:** Changes in arterial blood gas parameters with HFNC HFNC: high-flow nasal cannula oxygen; COPD: chronic obstructive pulmonary disease; PaCO2: partial carbon dioxide; PaO2: partial pressure of oxygen in arterial blood; SpO2: peripheral oxygen saturation; HCO3: bicarbonate.

Variables [median (IQR)]		pH	PaO2 (mmHg)	PaCO2 (mmHg)	HCO3 (mmol/L)	Lactate (mmol/L)	SpO2 (%)
All Patients (n=120)	Before HFNC	7.4 (7.32-7.46)	54.3 (47.2-61.5)	36.9 (30.1-47.7)	22.6 (20.1-25.5)	2.5 (1.5-3.4)	87 (81-92)
	After HFNC	7.4 (7.33-7.46)	72.1 (63-97.1)	36.25 (29.8-44.5)	22.7 (20-26)	1.7 (1.2-2.7)	95 (92-98)
	p	0.44	<0.001	0.47	0.94	<0.001	<0.001
Hypercapnia (n=35)	Before HFNC	7.31 (7.25-7.35)	57.1 (47.4-69.4)	49.9 (48.5-55.7)	23.9 (21.5-28)	2.1 (1.4-3)	88 (78-94)
	After HFNC	7.34 (7.29-7.40)	69.8 (63-90.2)	46.3 (40.9-56.7)	23.7 (21.5-28)	1.5 (1.2-2.2)	95 (92-98)
	p	<0.001	<0.001	<0.001	0.37	0.018	<0.001
COPD (n=32)	Before HFNC	7.395 (7.33-7.43)	54.55 (47.4-61.6)	42.9 (30.9-50.4)	22.7 (20.8-25.9)	2.7 (1.4-3.7)	88 (84-91)
	After HFNC	7.375 (7.33-7.43)	72.6 (65.4-106)	40.1 (32.6-53.4)	23.75 (19.4-28.5)	1.3 (1.1-2.5)	95 (92-98)
	p	0.82	<0.001	0.68	0.46	<0.001	<0.001
Respiratory acidosis (n=27)	Before HFNC	7.3 (7.21-7.32)	56.2 (47.3-68.9)	49.9 (48.3-56.8)	22.5 (19.9-24.9)	2 (1.4-3.5)	87 (76-92)
	After HFNC	7.33 (7.28-7.32)	68.2 (62.4-87.3)	48 (40.3-58.2)	23.2 (21.3-26.4)	1.5 (1.1-2.4)	95 (91-98)
	p	<0.001	0.002	0.004	0.19	0.05	<0.001
High lactate (n=71)	Before HFNC	7.4 (7.32-7.46)	53.3 (47-60.6)	34.8 (29.1-44.8)	21.5 (19.3-24.6)	2.9 (2.6-4.4)	85 (80-90)
	After HFNC	7.39 (7.34-7.45)	72.2 (64.4-92)	34.9 (29.5-43.5)	22.2 (18.2-25.8)	2 (1.5-3.8)	95 (93-98)
	p	0.48	<0.001	0.65	0.74	<0.001	<0.001

## Discussion

Several studies have reported that HFNC improves PaCO2 and other ABG parameters but there is limited data of its use in acute settings such as ED. In this study, we aimed to evaluate the effects of HFNC on blood gas parameters in patients with respiratory failure in ED. While non-invasive mechanical ventilation is first-line treatment with hypercapnia, Sun J et al. reported that mild and moderate hypercapnic patients can be managed with HFNC [7]. HFNC improved PaCO2 in initially hypercapnic patients in our study which is concurrent with the results of a number of other studies [8-10]. Our results also suggest that hypercapnic patients can be managed with HFNC in ED.

High rates of oxygen therapy might increase the tendency to hypercapnia and its association with the poor outcome. In our study, COPD group had no significant change in PaCO2 levels. This could also be interpreted as HFNC does not increase PaCO2 values in patients prone to hypercapnia. Chronic hypercapnia patients were not differentiated from acute hypercapnia patients in our study, but as illustrated in Figure 2, most patients with hypercapnia had their PaCO2 reduced by HFNC, therefore, our data also suggests whether PaCO2 levels increased chronic or in acute settings, most patients with hypercapnia might benefit from HFNC in ED. COPD patients with hypercapnia occurring in chronic settings might not improve from HFNC utilization as much as hypercapnia occurring in acute settings. The reasoning behind these findings requires further investigation with further studies for better utilization of HFNC.

Our study has also shown an improvement in other ABG parameters such as PaO2, lactate SpO2, and pH values. Patients with initial respiratory acidosis had improved pH levels after treatment while patients with initial normal pH remained in expected parameters. These findings are similar to other studies reporting improved physiological parameters with HFNC in acute respiratory failure [11,12].

High flow oxygenation generates PEEP resulting in increased oxygenation and carbon dioxide clearance. HFNC systems can deliver a flow of up to 60 L/min. As stated, in our ED, we have a standard protocol that initiates HFNC at 30 L/min but the initial fraction of inspired oxygen (FiO2) may differ depending on individual patient needs. The reasoning behind such a high rate of initiating flow was ARF treatment in our facility starts with either supplemental oxygen or mechanical ventilation depending on patient admission conditions in our ED. Therefore in our ED, HFNC commonly used in patients who do not benefit from initial conventional oxygen administration and does not immediately require mechanical ventilation support. In this regard, our study is different from other studies initiating the oxygen support to ARF patients with HFNC on admission. The study conducted by Rittayamai et al. explored flow rates' effect on respiratory workload and also found that 30 lt/min had better outcomes than lower flow rates [13]. While other studies showing gradually increased flow rates might be beneficial, due to the retrospective method, we could not specify which patients might benefit from different rates of flow. Although we did not have different rates of flow data, higher rates of flow have been reported to increase PaCO2 washout [14]. The effects of different flow rates need exploring with future studies.

HFNC initiation after supplemental oxygen treatment also improves pH and PaO2 parameters in ARF patients. Jones et al. reported HFNC improves PaO2 better than supplemental oxygen treatment [15]. Additionally, we found that PaO2, lactate, and SpO2 improved in all groups while pH only improved in patients with initial respiratory acidosis or hypercapnia. This data is consistent with reports from several other studies [8,12,16].

This study had several limitations. Patients with ARF might be using oxygen therapy at home or received in EMS during transport. We also did not include other medical treatments patients received before ED such as inhalers, corticosteroids, etc. Therefore initial ABG results should not be interpreted as the baseline of the patients' condition but as baseline parameters before starting the HFNC.

Also, the time of second ABG parameters acquired from patients was not standardized therefore, sequential ABG parameters do not reflect the exact time interval of ABG difference for every patient.

We excluded the patients who required noninvasive or invasive ventilation between sequential ABGs to evaluate HFNC efficiency but this might also lead to selection bias.

This study was conducted at a single center; thus, the ability to generalize the results may be limited.

While HFNC had positive effects of several parameters in ABGs, in the future randomized controlled studies are required for understanding the benefits of HFNC in patients with hypercapnia that occurred in acute or chronic settings.

## Conclusions

Treatment with HFNC produced improvement of blood gas parameters in subjects with acute respiratory failure in ED. These results suggest that HFNC can be used in hypercapnic patients as well as hypoxemic patients. Further randomized controlled studies are required to establish the impact of HFNC in the ED.
